# Faecal carriage of antibiotic resistant *Escherichia coli* in asymptomatic children and associations with primary care antibiotic prescribing: a systematic review and meta-analysis

**DOI:** 10.1186/s12879-016-1697-6

**Published:** 2016-07-25

**Authors:** Ashley Bryce, Céire Costelloe, Claire Hawcroft, Mandy Wootton, Alastair D. Hay

**Affiliations:** 1 Centre for Academic Primary Care, NIHR School for Primary Care Research, School of Social and Community Medicine, University of Bristol, Canynge Hall, 39 Whatley Road, Bristol, BS8 2PS UK; 2 NIHR Health Protection Research Unit in Healthcare Associated Infections and Antimicrobial Resistance, Imperial College London, Hammersmith Campus, W12 0NN London, UK; 3 Specialist Antimicrobial Chemotherapy Unit, Public Health Wales Microbiology Cardiff, University Hospital of Wales, Heath Park, Cardiff, CF14 4XW UK

## Abstract

**Background:**

The faecal reservoir provides optimal conditions for the transmission of resistance genes within and between bacterial species. As key transmitters of infection within communities, children are likely important contributors to endemic community resistance. We sought to determine the prevalence of antibiotic-resistant faecal *Escherichia coli* from asymptomatic children aged between 0 and 17 years worldwide, and investigate the impact of routinely prescribed primary care antibiotics to that resistance.

**Methods:**

A systematic search of Medline, Embase, Cochrane and Web of Knowledge databases from 1940 to 2015. Pooled resistance prevalence for common primary care antibiotics, stratified by study country OECD status. Random-effects meta-analysis to explore the association between antibiotic exposure and resistance.

**Results:**

Thirty-four studies were included. In OECD countries, the pooled resistance prevalence to tetracycline was 37.7 % (95 % CI: 25.9–49.7 %); ampicillin 37.6 % (24.9–54.3 %); and trimethoprim 28.6 % (2.2–71.0 %). Resistance in non-OECD countries was uniformly higher: tetracycline 80.0 % (59.7–95.3 %); ampicillin 67.2 % (45.8–84.9 %); and trimethoprim 81.3 % (40.4–100 %). We found evidence of an association between primary care prescribed antibiotics and resistance lasting for up to 3 months post-prescribing (pooled OR: 1.65, 1.36–2.0).

**Conclusions:**

Resistance to many primary care prescribed antibiotics is common among faecal *E. coli* carried by asymptomatic children, with higher resistance rates in non-OECD countries. Despite tetracycline being contra-indicated in children, tetracycline resistance rates were high suggesting children could be important recipients and transmitters of resistant bacteria, or that use of other antibiotics is leading to tetracycline resistance via inter-bacteria resistance transmission.

**Electronic supplementary material:**

The online version of this article (doi:10.1186/s12879-016-1697-6) contains supplementary material, which is available to authorized users.

## Background

The global emergence of antibiotic resistant bacterial infections is arguably the greatest 21st century threat to human health. The reasons for its emergence are complex and likely to include interactions between: the way in which antibiotics are used, particularly in primary care, where 80 % of all health service antibiotics are prescribed [1]; patient misuse through suboptimal dosing and antibiotic storage for future symptoms; over-the-counter (OTC) use; and community contacts and transmission. The more antibiotics a population is exposed to, the easier it becomes for resistant bacteria to spread and persist within communities. As key transmitters of infection within communities [2], children are likely to be important contributors to endemic community resistance.

The faecal reservoir provides optimal conditions for the transmission of resistance genes within and between bacterial species. *E. coli* is among the most abundant organisms in the faecal flora, both in humans and animals. *E. coli* is an opportunistic pathogen, and a common cause of urinary tract, bloodstream, and foodborne infections, and a cause of meningitis in neonates [3]. Whilst antibiotic use is likely to be the main driver of selection pressure contributing to antibiotic resistance [4], previous research has also demonstrated that intestinal bacteria can acquire resistance to certain antibiotics in the absence of antibiotic exposure [5]. How this resistance is acquired is unclear, but could be as a result of person-to-person transmission or environmental acquisition of resistant bacteria.

There has been little research published exploring faecal carriage of bacterial resistance in any asymptomatic population. This could provide important information regarding carriage and transmission of resistant bacteria within and between populations. This is particularly important in low-income countries, where antibiotics are often available OTC, without the need for a prescription [6]. Misuse of antibiotics in this way can expose harmless or opportunistic bacteria to a plethora of antibiotics to which they develop resistance. We conducted a systematic review aimed to investigate the carriage of faecal *E. coli* from asymptomatic children resistant to commonly prescribed primary care antibiotics, and quantify the relationship between previous exposure to primary care antibiotics and bacterial resistance. We stratified data by study country Organisation for Economic Co-operation and Development (OECD) status, as antibiotics can often be used differently in these population groups; antibiotics are obtained mostly by prescription only in OECD countries, whereas in non-OECD countries many antibiotics can be obtained over-the-counter [7–11].

## Methods

### Search strategy and selection criteria

We searched Medline, Embase, Cochrane and ISI Web of Knowledge databases for articles published in any language between 1940 and September 2015. MeSH terms for these databases included “drug resistance”, “faeces”, “carrier state” and “children”. MeSH terms were combined with text word searches which included “antibiotics”, “resistance”, “faecal/fecal”, “colonisation”, “commensal” and “paediatric/pediatric”. Grey and unpublished literature was searched for using ISI Web of Knowledge software and included journal articles, websites, conference proceedings, government and national reports and open access material. Reference lists of selected key papers were screened and authors who appeared multiple times in our search were contacted to request details of further published and unpublished work. All full-text papers were subject to citation searches. See Additional file 1 for full search strategy. The review protocol is available on PROSPERO (http://www.crd.york.ac.uk/PROSPERO/), registration number CRD42014009691.

Two independent reviewers screened all titles and abstracts for eligibility. Studies were eligible if they met the following criteria: investigated and reported carriage of resistance in faecal *E. coli* from asymptomatic children, that is children who were not showing symptoms of infection at the time the sample was taken; or investigated associations between previous antibiotic exposure and carriage of resistant *E. coli*; and study participants were children aged 0–17 years, including healthy neonates with an uncomplicated vaginal birth.

### Data extraction and quality assessment

Full-text papers for all eligible studies were obtained and three reviewers extracted data independently using a purpose-built spreadsheet. The following information was extracted from each paper, where provided: author, journal, year of publication, study design, study country, economic status, participants and recruitment location, recruitment time period, age range, method of faecal sample collection and testing, method of antimicrobial sensitivity testing, bacteria cultured and reported antibiotic sensitivities, previously prescribed antibiotics and time between antibiotic exposure and faecal sample collection. Economic status was measured using the OECD status of the country where the study was conducted [12]. The OECD is an international economic organisation first established in 1948, now made up of 34 countries, which aims to work together and with emerging and developing economies to reduce poverty through economic growth and financial stability [12]. OECD member countries tend to be ‘developed’ countries, whereas non-OECD countries tend to be ‘developing’. For the purpose of this review, we use OECD status as a general measure of country-level development, and a proxy marker for OTC antibiotic use. For antimicrobial exposure, time was generally recorded as a period of days, weeks or months prior to the faecal sample being taken and resistance being measured when the child had been exposed to any, or specific named antibiotics. Where any information was unclear in the paper, authors were contacted for clarification.

We reported resistance to antibiotics commonly prescribed to children in primary care, including for urinary tract infection or other indications including respiratory and skin infections. Resistance data was extracted and reported for the following antibiotics: ampicillin, co-amoxiclav (amoxicillin-clavulanic acid), co-trimoxazole (trimethoprim-sulfamethoxazole), trimethoprim, nitrofurantoin, ciprofloxacin, ceftazidime, tetracycline and chloramphenicol. Ceftazidime was the most frequently reported of all first to third generation cephalosporins, and acts as a marker for cephalosporin resistance.

Included papers were assessed for quality using a checklist based on Cochrane collaboration’s ‘risk of bias’ tool [13], We focused our quality criteria on factors we considered important for the review, namely: a reliable measure of antibiotic exposure and resistance, clear reporting of bacterial resistance, and clear reporting of children as asymptomatic or non-infected. For papers which included information on previous antibiotic exposure, we supplemented these with assessment of reporting adjustment for confounders including age, sex and socioeconomic status.

### Data synthesis and analysis

All statistical analyses were conducted using STATA version 13 software, and all methods undertaken according to PRISMA guidelines [14].

We calculated pooled prevalence of resistance estimates by generating a Forest plot for each antibiotic, stratified by OECD status. Forest plots illustrated proportion of resistant *E. coli* for each country, along with 95 % confidence intervals (CI), and the pooled prevalence of resistance per antibiotic per economic country group (OECD vs. non-OECD). We calculated pooled estimates for each country and for OECD and non-OECD groups using the pooled country estimates. Pooled prevalence estimates were generated for children of all age groups (0–90 days, 0–5 years and 5–17 years) and for defined time periods (1970–1979, 1980–1989, 1990–1999, 2000–2010, 2010–2015), for comparison. An I^2^ of 25, 50 and 75 % were used to signify low-level, moderate-level and high-level heterogeneity, in line with Cochrane recommendations [13]. All pooled estimates and 95 % confidence intervals (CI) were generated using double arcsine transformation to adjust for variance instability. This avoids implausible 95 % CI for prevalence estimates when generated under the normal approximation [15].

For studies investigating the association between previous antibiotic exposure and bacterial resistance, the outcome measure was the odds ratio (OR) of bacterial resistance in children previously exposed to any antibiotic compared to those children who were previously unexposed. The crude estimates from these studies were grouped according to the reported preceding exposure time period (0–2 weeks, 0–1 month and 0–3 months). A random-effects meta-analysis was conducted where heterogeneity was moderate-to-high and a pooled OR was generated for each exposure time period measured. These were compared to adjusted OR for each time period, where reported. Variables which were adjusted for were family member antibiotic exposure, previous hospitalisation, day care attendance, nappy use, ethnicity and socio-economic status (see Additional file 2). We assessed heterogeneity using the I^2^ statistic, and the null hypothesis of no heterogeneity was tested using the Q statistic generated from the *χ*^2^ test. Finally, funnel plots were generated to explore the possibility of small study effects, which can be caused by publication bias.

## Results

### Study characteristics

**Fig. 1:**
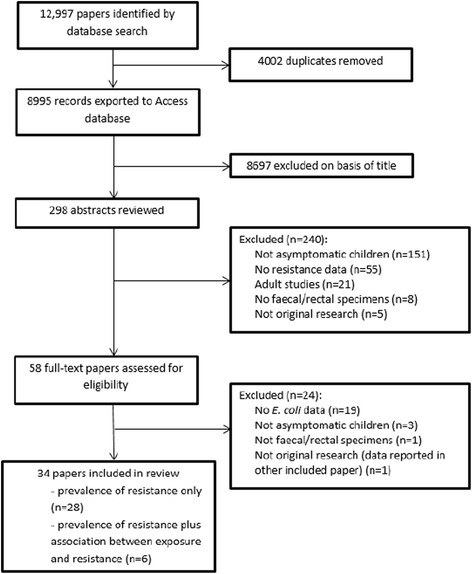
Data search and extraction (PRISMA flowchart)

We initially identified 12,997 potentially eligible articles. Of these, 8995 non-duplicate papers were assessed and 8697 excluded on basis of title (Fig. 1). The remaining 298 papers were assessed by abstract screening of which 240 were excluded. For the remaining 58, full-text papers were assessed, with 24 papers excluded. Thirty-four papers were therefore included in the review [16–49], of which six papers reported previous antibiotic exposure data and were included in our meta-analysis [19, 27, 33, 34, 37, 49].

**Fig. 2:**
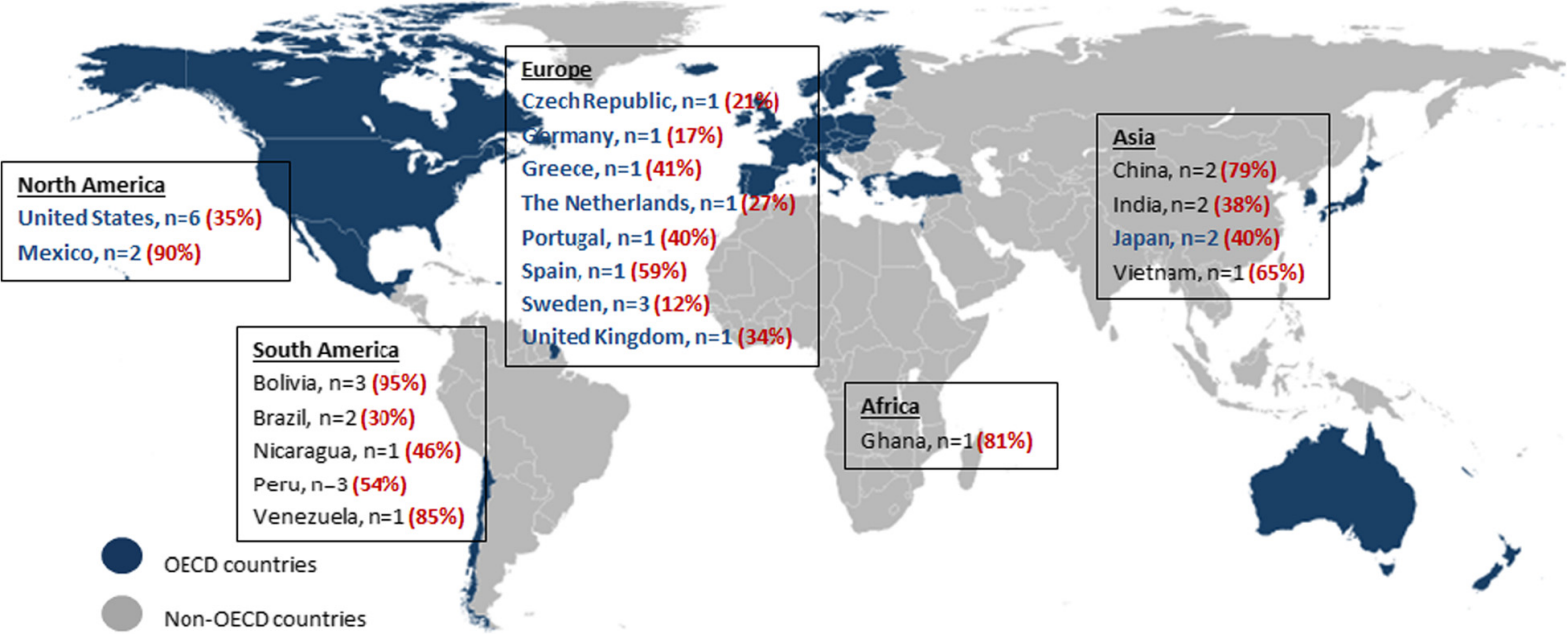
Geographical distribution of OECD and non-OECD countries, including number of included studies per country^a^ (OECD countries shown in *blue*) [12]. Faecal carriage of *E. coli* resistant to ampicillin for each reporting country are shown in *red*. Authors own map. ^a^ One study was conducted in the USA, but also reported resistance data from Venezuela and China, this study therefore appears three times [29]

Table 1 summarises the characteristics of the 34 studies. Additional study characteristics can be found in Additional file 2. Twenty studies, reporting the resistance status of 3864 *E. coli* isolates were conducted in OECD countries (Fig. 2), and all were observational. Fourteen studies, reporting the resistance status of 6699 isolates were conducted in non-OECD studies (Fig. 2), and again, all were observational. Twenty-three studies (14 OECD vs. 9 non-OECD) reported stool sampling as the primary collection method, with 10 reporting rectal swabs, and one study accepting both methods of collection. Antimicrobial sensitivity testing was carried out using standard disk diffusion methods for all studies, which were interpreted and reported according to either the European Committee on Antimicrobial Susceptibility Testing (EUCAST) [50], or the Clinical and Laboratory Standards Institute (CLSI) [51]. All study participants were healthy, without symptoms of infection and recruited in the community, schools and day care centres, or at a primary care facility conducting routine child health surveillance check-ups. Three papers, OECD only, included healthy neonates following uncomplicated vaginal delivery recruited from maternity units.

**Table 1:** Study characteristics of included papers

Study Characteristics	OECD (*n* = 20)		Non-OECD (*n* = 14)	
	Number of papers	Reference number	Number of papers	Reference number
Study Design:				
Retrospective observational	12	[16–27]	10	[36–45]
Prospective observational	5	[28–32]	1	[46]
Cross-sectional	3	[33–35]	3	[47–49]
Number of study participants:				
0–50	1	[21]	1	[38]
51–100	5	[25, 28, 30, 31, 34]	2	[42, 46]
101–200	7	[17, 18, 20, 22, 23, 29, 32]	2	[36, 40]
201–500	4	[24, 27, 33, 35]	2	[41, 43]
501–1000	3	[16, 19, 26]	5	[37, 39, 44, 47, 49]
1000+	0		2	[45, 48]
Method of faecal sampling:				
Stool sample	14	[17, 19–21, 23, 24, 27, 28, 30–35]	9	[36, 37, 39–41, 44, 46–48]
Rectal swab	5	[16, 18, 22, 25, 26]	5	[38, 42, 43, 45, 49]
Stool sample or rectal swab	1	[29]	0	
Age range of children^a^:				
Neonates (0–90 days)	3	[30–32]	0	
0–5 years	9	[18–21, 24, 28, 33–35]	7	[36, 37, 39, 41, 46, 48, 49]
5–17 years	4	[16, 23, 24, 26]	3	[40, 42, 44]
0–17 years	5	[17, 22, 25, 27, 29]	4	[38, 43, 45, 47]
Antibiotics reported:				
Ampicillin	15	[16–19, 21, 22, 24–26, 29–32, 34, 35]	12	[36–45, 47]
Co-amoxiclav	5	[16, 17, 19, 21, 28]	2	[39, 47]
Co-trimoxazole	4	[16, 17, 19, 21]	10	[36–41, 43–45, 47]
Trimethoprim	7	[22, 26, 28, 29, 33–35]	2	[29, 42]
Nitrofurantoin	3	[26, 30, 35]	2	[43, 48]
Ciprofloxacin	4	[16, 17, 21, 27]	12	[37–43, 45–48]
Ceftazidime	4	[16–18, 21]	3	[36, 39, 47]
Tetracycline	13	[16, 17, 20, 21, 23–26, 29–32, 35]	10	[36–38, 40–45, 47]
Chloramphenicol	11	[16, 17, 21, 23, 25, 26, 29–32, 35]	10	[37–39, 41–45, 47, 48]
Previous exposure to antibiotics^b^:				
0–2 weeks	2	[33, 34]	0	
0–3 weeks	0		1	[37]
0–1 month	1	[19]	0	
0–3 months	1	[27]	1	[49]
0–6 months	0		0	
0–1 year	0		0	

^a^Age 0–5 years: papers which report data specifically for this age group, 6–17 years: papers which report data specifically for this age group; 0–17 years: papers which report data for the children within 0–17 years, and do not fit into the previous reported age groups. Papers may appear more than once depending on how they have reported their results

^b^Indicates papers which reported information regarding previous exposure to antibiotics and the exposure time periods they investigated

The quality assessment ‘traffic-light’ charts for the included studies show that, for the six studies reporting antibiotic exposure information, reporting was generally good for our all our key quality indicators (Additional file 3). For studies reporting prevalence of resistance only, overall quality was good with the exception of controlling for confounding and accurate reporting of methods of analysis.

### Prevalence of resistance in faecal *E. coli* from asymptomatic children

Figure 2 details the number of studies per country and shows the global variation in resistance to ampicillin by OECD status. Resistance to ampicillin in faecal *E. coli* from asymptomatic children was highest in Mexico (OECD) and Bolivia (non-OECD), with a pooled prevalence of 90 and 95 %, respectively. Ampicillin resistance was lowest in Sweden (OECD), with a pooled prevalence of 12 %.

Table 2 shows the pooled prevalence of resistance to antibiotics in faecal *E. coli* isolates and were obtained from Forest plots generated for each antibiotic, which can be found in Additional files 4, 5, 6, 7, 8, 9, 10, 11 and 12.

**Table 2:** Pooled prevalence (%) of resistance to antibiotics in faecal *E. coli* from asymptomatic children (see Additional files 4, 5, 6, 7, 8, 9, 10, 11 and 12 for corresponding Forest plots)

Antibiotics	OECD					Non-OECD				
	Pooled prevalence (95 % CI)	Number of isolates tested	Number of studies	I^2^	Ref No.	Pooled prevalence (95 % CI)	Number of isolates tested	Number of studies	I^2^	Ref No.
Tetracycline	37.7 % (25.9–49.7 %)	2438	13 (9 countries)	19.5 %	[16, 17, 20, 21, 23–26, 29–32, 35]	80.0 % (59.7–95.3 %)	5401	10 (8 countries)	62.9 %	[36–38, 40–45, 47]
Ampicillin	37.6 % (24.9–54.3 %)	3053	15 (11 countries)	34.6 %	[16–19, 21, 22, 24–26, 29–32, 34, 35]	67.2 % (45.8–84.9 %)	6139	12 (10 countries)	58.1 %	[36–45, 47]
Trimethoprim	28.6 % (2.2–71.0 %)	862	7 (4 countries)	47.2 %	[22, 26, 28, 29, 33–35]	81.3 % (40.4–100 %)	359	2 (3 countries)	50.3 %	[29, 42]
Chloramphenicol	13.4 % (5.2–24.0 %)	2224	11 (8 countries)	58.1 %	[16, 17, 21, 23, 25, 26, 29–32, 35]	44.5 % (24.1–66.6 %)	5665	10 (9 countries)	39.0 %	[37–39, 41–45, 47, 48]
Co-trimoxazole	10.8 % (5.7–16.7 %)	1007	4 (4 countries)	33.5 %	[16, 17, 19, 21]	59.5 % (31.3–85.7 %)	5439	10 (6 countries)	61.4 %	[36–41, 43–45, 47]
Co-amoxiclav	7.2 % (1.8–13.5 %)	1066	5 (5 countries)	37.9 %	[16, 17, 19, 21, 28]	18.1 % (14.7–21.6 %)	745	2 (2 countries)	8.8 %	[39, 47]
Ciprofloxacin	5.1 % (0.2–17.8 %)	586	4 (4 countries)	36.0 %	[16, 17, 21, 27]	9.3 % (3.4–17.2 %)	6231	12 (7 countries)	60.3 %	[37–43, 45–48]
Nitrofurantoin	4.4 % (0.6–9.3 %)	673	3 (3 countries)	54.4 %	[26, 30, 35]	7.3 % (5.9–9.7 %)	715	2 (2 countries)	0.0 %	[43, 48]
Ceftazidime	0.3 % (0.1–0.8 %)	177	4 (4 countries)	0.0 %	[16–18, 21]	5.0 % (0.7–15.6 %)	654	3 (3 countries)	28.8 %	[36, 39, 47]

Ordered by pooled resistance prevalence in OECD countries (highest to lowest)

In OECD countries, the highest pooled resistance prevalence was for tetracycline at 37.7 % (95 % CI: 25.9–49.7 %), with ampicillin and trimethoprim resistance also high at 37.6 and 28.6 %, respectively. Resistance to ceftazidime was lowest in OECD countries at 0.3 % (0.1–0.8 %).

Similarly to OECD countries, in non-OECD countries the highest pooled prevalence of resistance was observed in the same antibiotics, with trimethoprim highest at 81.3 % (95 % CI: 40.4–100 %), followed by tetracycline and ampicillin at 80.0 and 67.2 %, respectively.

### Prevalence of resistance in different age groups

There were too few data to report pooled resistance prevalence estimates for any given age group (0–90 days, 0–5 years, 5–17 years) for any antibiotic reported in this review.

### Prevalence of resistance across different time periods

**Fig. 3:**
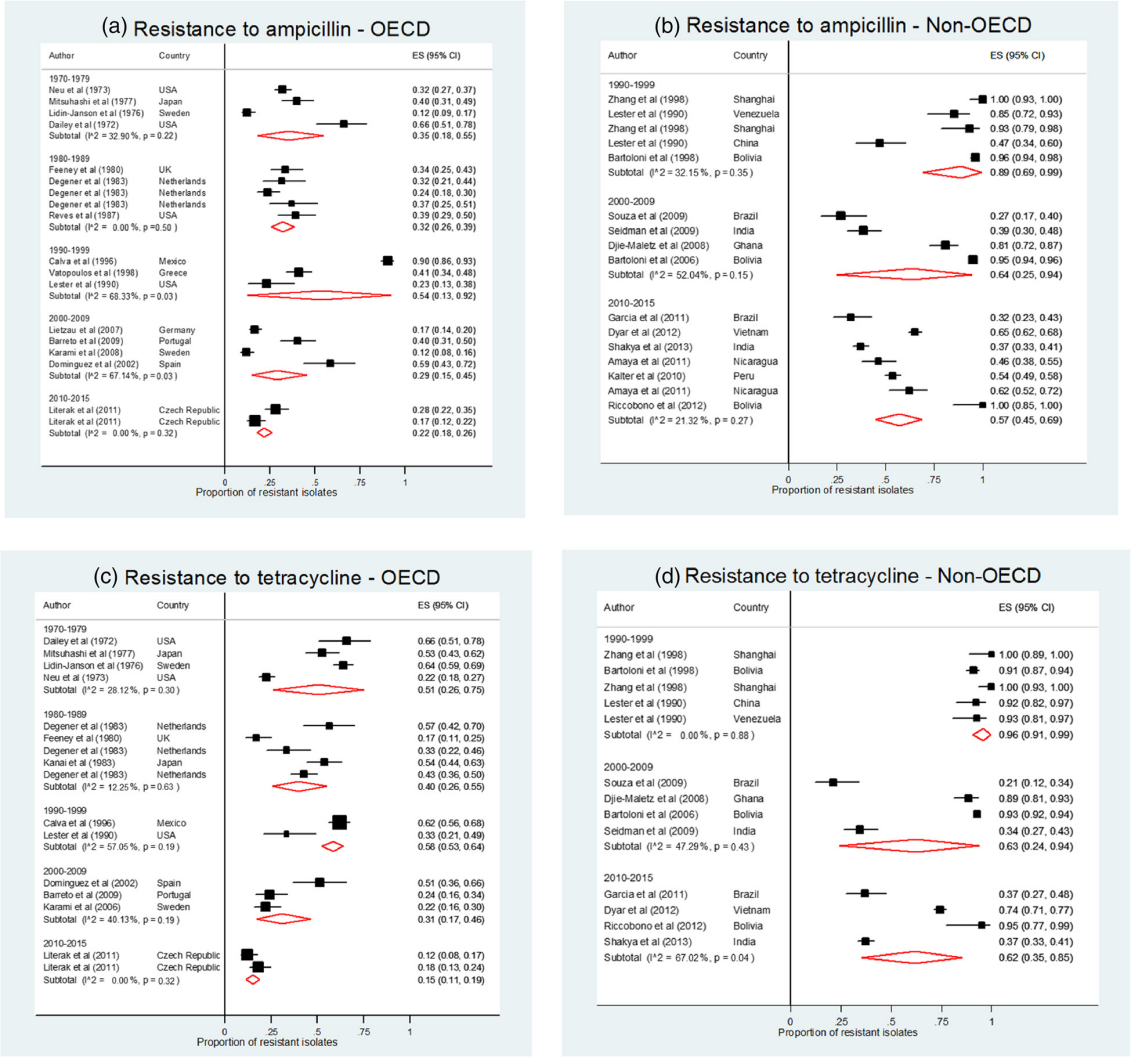
Pooled prevalence (%) of resistance to antibiotics in faecal *E. coli* from asymptomatic children across different time periods for ampicillin (**a** and **b**) and tetracycline (**c** and **d**), split by OECD (**a** and **c**) and non-OECD (**b** and **d**) countries. Studies included more than once reported resistance separately in different age groups or different geographic locations

Figure 3 shows a Forest plot of the pooled resistance prevalence to ampicillin and tetracycline (for which data were most complete), by OECD status, by decade. There were too few data for all other antibiotics to report time period estimates. For OECD countries, included studies were conducted between 1970 and 2014, compared with non-OECD countries which were conducted from 1990 to 2014. Once again, the graphs show the higher resistance rates in non-OECD compared to OECD countries, however there is no evidence of a change in resistance over time as the confidence intervals for each time period and each antibiotic overlap.

### Association between previous antibiotic exposure and bacterial resistance

**Fig. 4:**
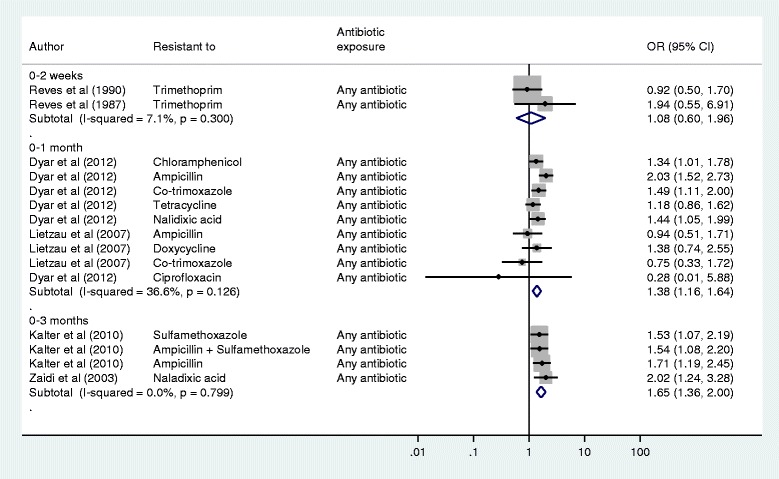
Meta-analysis of individual studies examining association between previous primary care antibiotic exposure and carriage of bacterial resistance. The Forest plot shows pooled crude and individual OR (log scale) for resistance in asymptomatic children’s faecal *E. coli* bacteria and previous exposure to any antibiotic. Studies grouped according to time period during which exposure was measured and ordered within each time period by increasing standard error

Figure 4 shows a Forest plot of six studies investigating the relationship between previous exposure to antibiotics and resistance to a range of commonly used primary care antibiotics. Within all antibiotic exposure time periods, the crude odds of resistance were generally greater for children exposed to antibiotics than those unexposed, though exposure at 0–2 weeks was not found to be significantly associated with resistance. The effect sizes are reasonably similar for all time periods, with the pooled OR of resistance rising as the cumulative antibiotic exposure period increases, though confidence intervals do overlap between different time periods. Given the overlap in exposure time periods, meta-regression analysis was not appropriate.

There was no evidence of within group heterogeneity in the 0–3 month time period, with low heterogeneity in the 0–2 week period and moderate heterogeneity in the 0–1 month periods. For those studies which reported adjusted ORs, adjusting for family member antibiotic exposure, previous hospitalisation, day care attendance, nappy use, ethnicity and socio-economic status; we compared these results with our crude estimates, though we only had sufficient data to do this for exposure at 0–3 months. The pooled adjusted (OR 1.70, 95 % CI: 1.36–2.12) and crude (OR 1.65, 1.36–2.00) did not differ substantially.

### Publication bias

There were too few studies for any given exposure time period to assess publication bias.

## Discussion

### Principal findings

In asymptomatic children, we found evidence of high rates of faecal *E. coli* resistance to several commonly prescribed primary care antibiotics, and we have shown that resistance rates were consistently higher in non-OECD compared to OECD countries. The routine use of primary care antibiotics could be an important contributor to carriage of resistant *E. coli* which we showed persists at both 1 and 3 months post-antibiotic prescription.

### Strengths and weaknesses

To our knowledge, this is the first systematic review and meta-analysis to explore and report global evidence regarding faecal carriage of resistant bacteria in healthy, community-resident children and associations with the routine use of antibiotics in primary care. Our review was rigorously conducted according to the Cochrane guidelines for Systematic Reviews [13]. We chose to stratify our results by OECD status to reflect both national development and likely OTC antibiotic availability [6, 52].

We are aware of four main limitations. First, antibiotics are used very differently within OECD and non-OECD countries [53–56], and OTC antibiotic use is difficult to measure. A systematic review conducted in 2011 reported high non-prescription antibiotic variability across countries worldwide [52], and there is not 100 % agreement between OECD status and OTC antibiotic availability. However, we are not aware of a better country-level alternative with respect to measuring global prevalence of antibiotic resistance in relation to antibiotic use, and none of the included studies reported or measured OTC antibiotic availability. There was some variation in heterogeneity for our pooled prevalence of resistance estimates. Most heterogeneity was moderate at around 50 %, higher heterogeneity was observed most frequently in our estimates from non-OECD countries. This may be due to the lack of information provided regarding the study populations; although all children were asymptomatic of infection, they may vary in other factors from country to country, for example the comorbidities. Higher heterogeneity in non-OECD countries may also be a reflection of the availability of certain antibiotics OTC in some countries. We also acknowledge that factors other than antibiotic usage and OTC availability can account for differences in carriage of resistant bacteria between OECD and non-OECD countries, including; poverty, poor sanitation, unstable governance, and lower levels of medicine regulation [57]. Additionally, the majority of our non-OECD studies were conducted in either South America or Asia, with African countries under-represented in this group.

Second, reverse causality and other confounding associations including age, sex and previous hospitalisation, could have introduced bias to our meta-analysis findings. However, analyses adjusting for confounding factors did not demonstrate substantial differences between crude and adjusted association estimates. Third, our meta-analysis of the association between antibiotic exposure and resistance reported moderate heterogeneity within the 0–1 month time period, however the difficulty in estimating a more accurate point of antibiotic exposure may have accounted for this. In addition to this, only six studies, conducted between 1987 and 2012 reported data on antibiotic exposure, therefore these findings must be interpreted with caution, but nevertheless provide some evidence to support the possibility of an association between antibiotic exposure and carriage of resistant bacteria. Finally, there were insufficient studies to adequately assess publication bias.

### Results in the context of existing research

#### Carriage of resistant faecal *E. coli* in healthy children

Resistance to ampicillin in faecal *E. coli* isolates was high for both OECD and non-OECD countries, particularly non-OECD countries which reached almost 65 %. There is little data other than that which was included in this review with which to compare estimates. However, the highest reported resistance to ampicillin was very similar to reported aminopenicillin group resistance in the European Antimicrobial Resistance Surveillance Network (EARS-Net) database and US Centre for Disease Dynamics, Economics and Policy (CDDEP) databases [58, 59]. Given that such databases include clinical samples from the general population, including older adults, the similarities observed here could be a result of between age-group transmission of genetic resistance factors such as plasmids; facilitated via frequent interaction between children and adults. In addition, the EARS-Net and CDDEP databases constitute ‘invasive’ clinical *E. coli* samples, taken from blood or urine. This could suggest that the resistance profiles of both commensal and pathogenic organisms are similar. A recent systematic review exploring prevalence of resistance to antibiotics in *E. coli* causing urinary tract infection in children also reported similar estimates to faecal *E. coli* [60], which further supports this theory.

Tetracycline can be used for a number of indications, but is not recommended for use in children under 8 years due to its association with permanent tooth discolouration [61]. Despite this, the pooled resistance prevalence to tetracycline was high in faecal *E. coli* from healthy children in both OECD and non-OECD countries. Previous studies in human faecal bacteria have reported that bacteria such as *E. coli* which are resistant to tetracycline also tend to be co-resistant to other antibiotics, including ampicillin and sulphonamides [62]. A UK study reported that following administration of amoxicillin in healthy adults, an increase in tetracycline resistance genes was observed in *E. coli* faecal isolates, an indication of co-selection of multiple antibiotic resistance genes [63]. The reason for the high-level resistance to tetracycline in asymptomatic children may not necessarily reflect exposure in individual children, but exposure from their contacts; indicating that community-level exposure to antibiotics may play a greater role in the dissemination of resistant bacteria than individual exposure in children. Additionally, there is considerable evidence demonstrating the transfer of resistance genes between animals and humans, whether through direct contact with animals such as pets [64], or through the ingestion of animal food-products [65]. Whilst the use of antibiotics, including tetracycline, as growth promoters in food animals is no longer recommended in many European countries, transfer of resistant bacteria in this manner continues to pose a global threat [66], as does veterinary use of antibiotics, which is less well regulated than human use.

#### Association between antibiotic exposure and carriage of resistant *E. coli*

Our meta-analysis of the association between previous exposure to antibiotics and bacterial resistance observed associations which were stronger for longer time periods, namely 0–1 month and 0–3 months compared with 0–2 weeks. There was no association found between antibiotic exposure within 0–2 weeks and carriage of resistance; this may have been due to insufficient sample size, or the fact that the studies measuring association within this time period were almost 30 years old, whereas the studies measuring associations in other time periods were more recent. Of the six studies included in our meta-analysis, most reported the association between previous antibiotic exposure and resistance within overlapping time periods. This implies that the associations with longer time periods (i.e. 0–3 months) could reflect either long or short-term relationships. A previous systematic review demonstrated similar effects in urinary and respiratory bacteria, in patients of all ages [67]. That review found that the effect of antibiotic exposure on the isolation of a resistant isolate may persist for up to 12 months, something we were unable to explore because our review studies did not measure exposure for this period.

#### Clinical, public health and research implications

Our findings demonstrate the high-level resistance to some of the most commonly prescribed primary care antibiotics in faecal isolates from healthy children, and suggest that one cause of carrying bacterial resistant faecal flora in healthy children could be previous exposure to antibiotics. Despite our data being obtained from asymptomatic children, the clinical and public health implications of these findings are significant. First, they provide further empirical data to support the importance of antimicrobial stewardship and good sanitation, and that the more antibiotics are prescribed and used within communities, either in humans, food products or farm animals and pets, the greater the selection pressure is for resistance to develop and persist. Resistant bacteria can be shed from humans and animals in faeces which can contaminate the environment, including water supplies. Second, faecal bacteria have been shown to be the source of auto-infection, in which safely carried bacteria invade other body areas and become pathogenic, leading to UTI, meningitis, septicaemia and pneumonia [3]. Autoinfection of resistant bacteria could result in the ineffectiveness of first-line antibiotic treatments, and without the development of any new antibiotics, this poses hazardous limitations on our continued ability to treat. For primary care clinicians, the best course of action is to consider the impact of any antibiotic use on antimicrobial resistance, and avoid their unnecessary use by following local and national guidance wherever possible.

Future studies should identify the extent of faecal shedding and modes of antibiotic-resistant bacteria transmission within and between communities of humans, animals and the surrounding environments.

## Conclusions

Resistance to many commonly used primary care antibiotics in faecal *E. coli* isolates from asymptomatic children ranged from moderate to very high, with resistance being higher in non-OECD countries. Routine antibiotic use is likely to be an important contributor to resistance, which may persist for up to 3 months post-antibiotic treatment. Despite the fact that tetracycline is contra-indicated in children, the high rates of tetracycline resistance suggest healthy children could be important recipients and transmitters of resistant bacteria and, or, that use of other antibiotics is leading to tetracycline resistance via inter-bacteria resistance transmission.

## Abbreviations

CDDEP, US centre for disease dynamics, economics and policy; CI, confidence interval; CLSI, clinical and Laboratory Standards Institute; EARS-net, European antimicrobial resistance surveillance network; EUCAST, European committee on antimicrobial sensitivity testing; OECD, Organisation for Economic Cooperation and Development; OR, odds ratio; OTC, over the counter

## Additional files

Additional file 1: Medline and Embase Search Strategy. (DOCX 13 kb) Additional file 2: Study characteristics table. (DOCX 18 kb) Additional file 3: Data quality charts (split by studies reporting prevalence of resistance only and prevalence plus antibiotic exposure). (DOCX 77 kb) Additional file 4: Ampicillin resistance in faecal *E. coli* isolates from asymptomatic children, by OECD status. (DOCX 160 kb) Additional file 5: Co-amoxiclav resistance in faecal *E. coli* isolates from asymptomatic children, by OECD status. (DOCX 108 kb) Additional file 6: Co-trimoxazole resistance in faecal *E. coli* isolates from asymptomatic children, by OECD status. (DOCX 144 kb) Additional file 7: Trimethoprim resistance in faecal *E. coli* isolates from asymptomatic children, by OECD status. (DOCX 96 kb) Additional file 8: Nitrofurantoin resistance in faecal *E. coli* isolates from asymptomatic children, by OECD status. (DOCX 89 kb) Additional file 9: Ciprofloxacin resistance in faecal *E. coli* isolates from asymptomatic children, by OECD status. (DOCX 45 kb) Additional file 10: Ceftazidime resistance in faecal *E. coli* isolates from asymptomatic children, by OECD status. (DOCX 38 kb) Additional file 11: Tetracycline resistance in faecal *E. coli* isolates from asymptomatic children, by OECD status. (DOCX 165 kb) Additional file 12: Chloramphenicol resistance in faecal *E. coli* isolates from asymptomatic children, by OECD status. (DOCX 152 kb)
