# Supplementary material for: Faecal carriage of antibiotic resistant Escherichia coli in asymptomatic children and associations with primary care antibiotic prescribing: a systematic review and meta-analysis
Source: BMC Infect Dis. 2016 Jul 25;16:359. doi: 10.1186/s12879-016-1697-6 (PMC4960702; doi:10.1186/s12879-016-1697-6)
Supplement: Additional file 1: — Medline and Embase Search Strategy. (DOCX 13 kb) [file 12879_2016_1697_MOESM1_ESM.docx]

**Additional file 1 Medline and Embase Search Strategy**

| **MEDLINE and EMBASE search strategy** | |
| --- | --- |
| 1. exp. Feces/ mi [Microbiology] | 22. exp. Bacteria |
| 1. exp. Colon/ mi [Microbiology] | 23. exp. Enterobacteriaceae |
| 1. exp Carrier State/ ep [Epidemiology] | 24. exp. Escherichia coli |
| 1. exp Carrier State/ mi [Microbiology] | 25. exp. Enterococcus |
| 1. f?ecal carriage.tw | 26. exp. Gram-negative Bacteria |
| 1. f?ecal.tw | 27. organis$.tw |
| 1. stool.tw | 28. isolate$.tw |
| 1. commensal.tw | 29. bacteria.tw |
| 1. coloni#ation.tw | 30. exp. Child |
| 1. coloni#$.tw | 31. exp. Child, Pre-school |
| 1. flora.tw | 32. exp. Infant |
| 1. microflora.tw | 33. exp. adolescent |
| 1. intestinal.tw | 34. child.tw |
| 1. shedding.tw | 35. children.tw |
| 1. exp. Drug Resistance, Microbial | 36. childhood.tw |
| 1. exp. Anti-bacterial Agents/ tu [Therapeutic Use] | 37. p?ediatri$.tw |
| 1. exp. Microbial Sensitivity Tests | 38. 1 or 2 or 3 or 4 or 5 or 6 or 7 or 8 or 9 or 10 or 11 or 12 or 13 or 14 |
| 1. antibiotic$.tw | 39. 15 or 16 or 17 or 18 or 19 or 20 or 21 |
| 1. antimicrobial.tw | 40. 22 or 23 or 24 or 25 or 26 or 27 or 28 or 29 |
| 1. antimicrobial resistance.mp | 41. 30 or 31 or 32 or 33 or 34 or 35 or 36 or 37 |
| 1. resistan$.tw | 42. 38 and 39 and 40 and 41 |
