# Supplementary material for: Faecal carriage of antibiotic resistant Escherichia coli in asymptomatic children and associations with primary care antibiotic prescribing: a systematic review and meta-analysis
Source: BMC Infect Dis. 2016 Jul 25;16:359. doi: 10.1186/s12879-016-1697-6 (PMC4960702; doi:10.1186/s12879-016-1697-6)
Supplement: Additional file 2: — Study characteristics table. (DOCX 18 kb) [file 12879_2016_1697_MOESM2_ESM.docx]

**Additional file 2 Study characteristics table**

| **Study** | **Author** | **Country** | **Design** | **Recruitment** | **n** | **Age range** | **Sampling method** | **Antibiotic susceptibilities** | **Guidelines used to interpret sensitivities** | **Previous exposure to antibiotics reported?** | **Variables adjusted for in antibiotic exposure analysis** |
| --- | --- | --- | --- | --- | --- | --- | --- | --- | --- | --- | --- |
| **OECD** | | | | | | | | | | | |
| 16 | Literak (2011) | Czech Republic | RO | C | 177 | 0-17 yrs | RS | AMP, TET, STR, COT, CL, NA, CHL, COA, CIP, GEN, CFZ | NR | N | - |
| 17 | Barreto (2009) | Portugal | RO | C | 92 | 1-14 yrs | SS | AMP, COA, GEN, TOB, AMI, STR, TET, COT, NA, CIP, CHL, CFZ, CTX, AZT, CX, IMI | CLSI | N | - |
| 18 | Karami (2008) | Sweden | RO | C | 272 | 0-1 yr | RS | AMP, CD, CX, CFU, CFZ | EUCAST | N | - |
| 19 | Lietzau (2007) | Germany | RO | HCC | 492 | 0-4 yrs | SS | AMP, COA, PIP-TAZ, CPD, CFU, DOX, GEN, COT, NA, LEV | NR | Y | Parents/siblings with resistant *E. coli*, day care attendance, previous hospitalisation |
| 20 | Karami (2006) | Sweden | RO | C | 128 | 0-3 yrs | SS | TET | EUCAST | N | - |
| 21 | Dominguez (2002) | Spain | RO | N | SS | 0-2 yrs | SS | AMP, COA, TIC, CZ, CX, CTX, CFZ, AZT, IMI, NA, CIP, GEN, TOB, APR, KAN, STR, AMI, TET, CHL, COT, FOS | CLSI | N | - |
| 22 | Vatopoulos (1998) | Greece | RO | HCC | 181 | 0-6 yrs | RS | AMP, TRI | CLSI | N | - |
| 23 | Kanai (1983) | Japan | RO | S | 104 | 6+ yrs | SS | TET, CHL, STR, KAN, PEN | NT | N | - |
| 24 | Degener (1983) | Netherlands | RO | C | 281 | 0-17 yrs | SS | TET, AMP, SUL | NR | N | - |
| 25 | Neu (1973) | USA | RO | PD | 370 | 0-8 yrs | RS | AMP, STR, TET, CHL | NR | N | - |
| 26 | Lidin-Janson (1976) | Sweden | RO | C | 306 | 7-16 yrs | RS | SUL, TET, STR, CHL, AMP, NIT, NA, TRI | NR | N | - |
| 27 | Zaidi (2003) | Mexico | RO | DCC, S | 276 | 0-12 yrs | SS | NA, CIP | CLSI | Y | Family members antibiotic exposure, family member hospitalisation |
| 28 | Singh (1990) | USA | PO | DCC | 100 | 0-2 yrs | SS | TRI, CFU, CLX, COA, CL | CLSI | N | - |
| 29 | Lester (1990)^a^ | USA (Venezuela, China) | PO | HCC, N | 39 | 0-6 yrs | SS, RS | STR, TET, SUL, AMP, TRI, CHL, GEN, KAN | NR | N | - |
| 30 | Feeney (1980) | UK | PO | MC | 107 | 0-7 days | SS | AMP, CHL, NA, NEO, NIT, STR, TET | NR | N | - |
| 31 | Mitsuhashi (1977) | Japan | PO | MC | 110 | 0-1 mth | SS | TET, CHL, KAN, STR, AMP, | NR | N | - |
| 32 | Dailey (1972) | USA | PO | MC | 41 | 0-5 days | SS | AMP, CHL, CL, STR, GEN, KAN, NA, TET | NR | N | - |
| 33 | Reves (1990) | USA | CS | DCC | 203 | 0-2 yrs | SS | TRI | NR | Y | Age, nappy use, duration in day care, ethnicity |
| 34 | Reves (1987) | USA | CS | DCC | 79 | 0-2 yrs | SS | TRI, AMP | CLSI | Y | None |
| 35 | Calva (1996) | Mexico | CS | C | 260 | 0-2 yrs | SS | AMP, TRI, TET, CHL, GEN, NIT, NOR | CLSI | N | - |
| **Non-OECD** | | | | | | | | | | | |
| 36 | Garcia (2011) | Brazil | RO | C | 84 | 0-5 yrs | SS | AMI, AMP, AMP-SUL, CL, CFZ, CFX, LEV, GEN, TET, COT, IMI, PIP-TAZ | CLSI | N | - |
| 37 | Dyar (2012) | Vietnam | RO | C | 818 | 0-5 yrs | SS | TET, COT, AMP, CHL, NA, CIP | EUCAST | Y | None |
| 38 | Riccobono (2012) | Bolivia/Peru | RO | C | 21 | 0-6 yrs | RS | AMP, CFX, TET, CHL, STR, KAN, GEN, AMI, COT, NA, CIP | NR | N | - |
| 39 | Amaya (2011) | Nicaragua | RO | HCC | 91 | 0-5 yrs | SS | COA, AMP, CFZ, CHL, CFX, CIP, GEN, IMI, COT | CLSI | N | - |
| 40 | Seidman (2009) | India | RO | C | 119 | 5-10 yrs | SS | AMP, AZT, CIP, CFX, GEN, NA, TET, COT, CZ, CTX | CLSI | N | - |
| 41 | Djie-Maletz (2008) | Ghana | RO | C | 105 | 0-4 yrs | SS | AMP, AMP-SUL, PIP, PIP-TAZ, CFU, COT, GEN, TOB, AMI, TET, CIP, CHL | EUCAST | N | - |
| 42 | Zhang (1998) | Shanghai | RO | N, S | 84 | 5-11 yrs | RS | AMP, PIP, GEN, AMI, STR, CHL, TET, SUL, TRI, NOR, OFL, CIP | CLSI | N | - |
| 43 | Bartoloni (1998) | Bolivia | RO | C | 321 | 0-6 yrs | RS | AMI, AMP, CTX, CL, CHL, CIP, COT, GEN, NA, NIT, PIP, TET | NR | N | - |
| 44 | Souza (2009) | Brazil | RO | C | 52 | 5-10 yrs | SS | COT, AMP, TET, CL, STR, CHL | CLSI | N | - |
| 45 | Bartoloni (2006) | Peru/Bolivia | RO | C | 3174 | 0-6 yrs | RS | AMP, COT, TET, STR, CHL, NA, KAN, GEN, CIP, AMI, CFX | NR | N | - |
| 46 | Pons (2014) | Peru | PO | C | 46 | 0-1 yr | SS | NA, CIP | CLSI | N | - |
| 47 | Shakya (2013) | India | CS | C | 529 | 3-14 yrs | SS | TET, AMP, PIP, COA, PIP-TAZ, CIP, NOR, NA, COT, CX, CTX, CFZ, CFX, CF, IMI, GEN, AMI, CHL | CLSI | N | - |
| 48 | Kristiansson (2009) | Peru | CS | C | 394 | 0-5 yrs | SS | STR, CHL, NA, KAN, CIP, GEN, NIT | NR | N | - |
| 49 | Kalter (2010) | Peru | CS | C | 523 | 0-3 yrs | RS | AMP, CFX, CIP, SUL, | CLSI | Y | Age, socioeconomic status, home-raised chickens |

Where - = not applicable

Design: RO = retrospective observational; PO = prospective observational; CS = cross-sectional.

Recruitment: C = community; HCC = health care centre; N = nursery; S = school; PD = paediatrician; DCC = day care centre; MC = maternity clinic

Sampling method: SS = stool sample; RS = rectal swab

Antibiotic susceptibilities: AMI = amikacin; AMP = ampicillin; AMP-SUL = ampicillin-sulbactam; APR = apramycin; AZT = aztreonam; CD = cedadroxil; CF = cefepime; CFU = cefuroxime; CFX = ceftriaxone; CFZ = ceftazidime; CHL = chloramphenicol; CIP = ciprofloxacin; CL = cefalothin; CLX = cefalexin; COA = co-amoxiclav; COT = co-trimoxazole; CP = cefprozil; CPD = cefpodoxime; CTX = cefotaxime; CX = cefoxitin; CZ = cefazolin; DOX = doxycycline; FOS = fosfomycin; GEN = gentamicin; IMI = imipenem; KAN = kanamycin; LEV = levofloxacin; MER = meropenem; NA = naladixic acid; NET = netilmicin; NIT = nitrofurantoin; NOR = norfloxacin; OFL = ofloxacin; PEN = penicillin; PIP = piperacillin; PIP-TAZ = piperacillin-tazobactam; STR = streptomycin; SUL = sulfamethoxazole; TAZ = tazobactam; TEM = temocillin; TET = tetracycline; TIC = ticarcillin; TOB = tobramycin; TRI = trimethoprim

Guidelines: CLSI = Clinical & Laboratory Standards Institute; EUCAST = European Committee on Antimicrobial Susceptibility Testing; NR = not reported

^a^ Reference 27 was conducted in USA, but also collected data from Venezuela and China (both Non-OECD).
