# Supplementary material for: Faecal carriage of antibiotic resistant Escherichia coli in asymptomatic children and associations with primary care antibiotic prescribing: a systematic review and meta-analysis
Source: BMC Infect Dis. 2016 Jul 25;16:359. doi: 10.1186/s12879-016-1697-6 (PMC4960702; doi:10.1186/s12879-016-1697-6)

**Additional file** 3 Data quality charts (split by studies reporting prevalence of resistance only and prevalence plus antibiotic exposure)


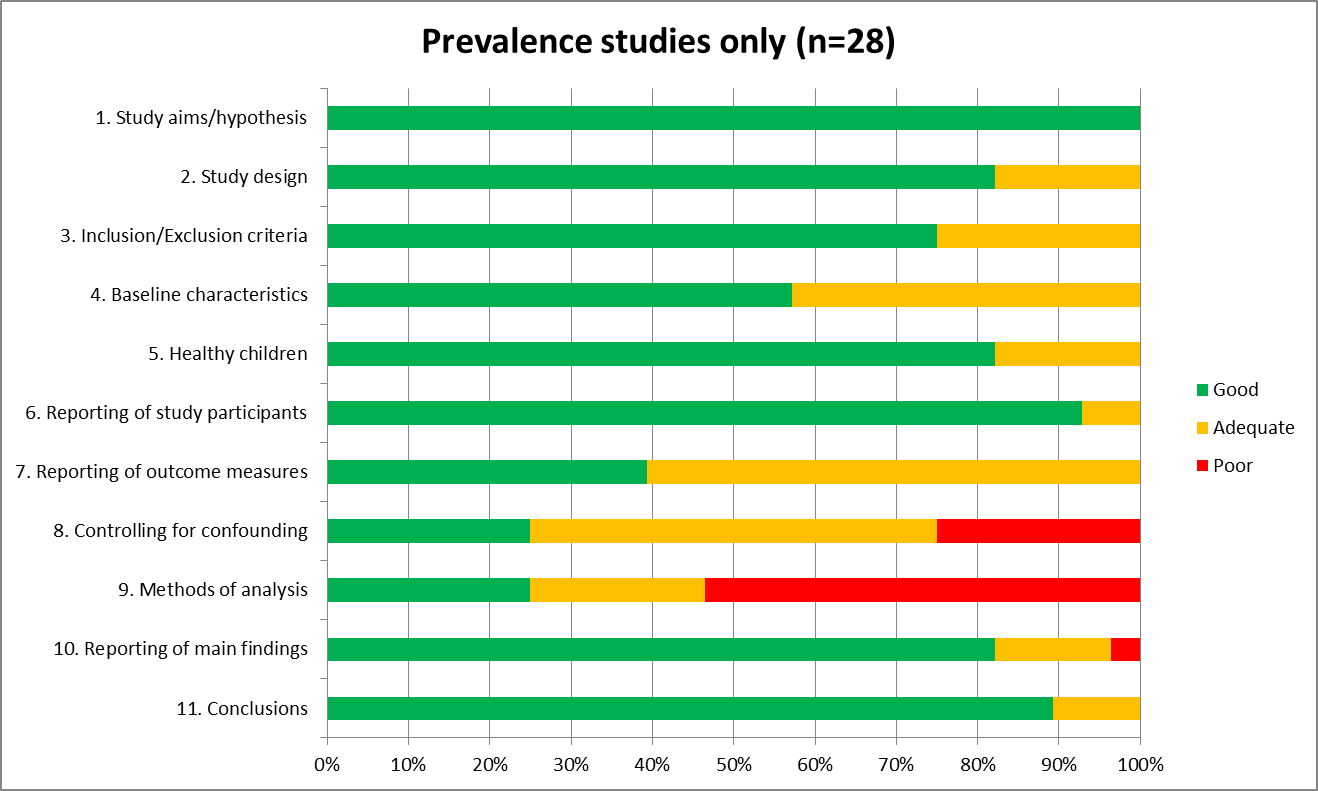

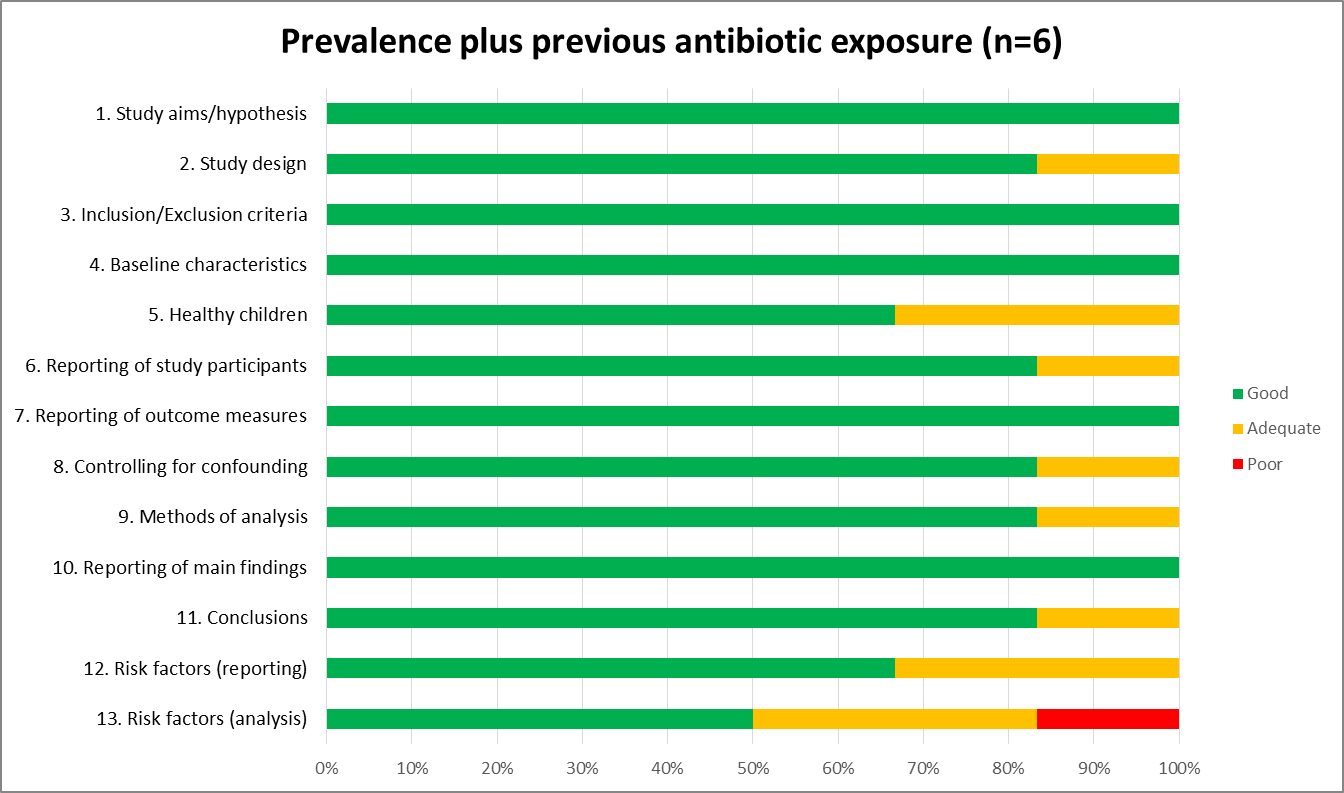

Supplement: Additional file 3: — Data quality charts (split by studies reporting prevalence of resistance only and prevalence plus antibiotic exposure). (DOCX 77 kb) [file 12879_2016_1697_MOESM3_ESM.docx]
