# Supplementary material for: Faecal carriage of antibiotic resistant Escherichia coli in asymptomatic children and associations with primary care antibiotic prescribing: a systematic review and meta-analysis
Source: BMC Infect Dis. 2016 Jul 25;16:359. doi: 10.1186/s12879-016-1697-6 (PMC4960702; doi:10.1186/s12879-016-1697-6)
Supplement: Additional file 11: — Tetracycline resistance in faecal E. coli isolates from asymptomatic children, by OECD status. (DOCX 165 kb) [file 12879_2016_1697_MOESM11_ESM.docx]

**Additional file 11 Tetracycline resistance in faecal *E. coli* isolates from asymptomatic children, by OECD status**


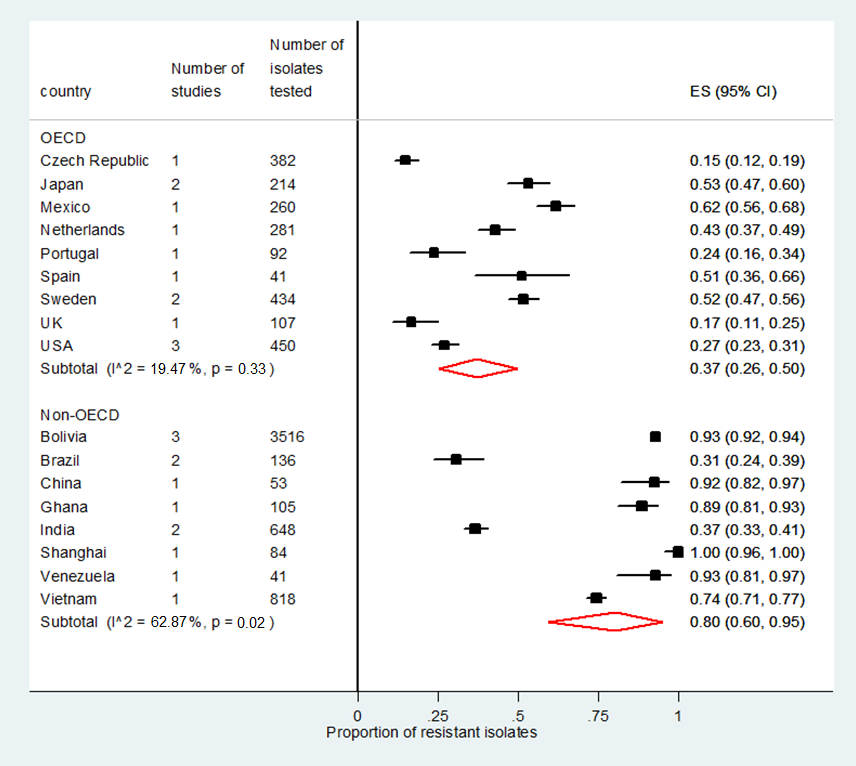
 Where ES = effect size
